# Phase I study to evaluate the maximum tolerated dose of the combination of SH003 and docetaxel in patients with solid cancer: A study protocol

**DOI:** 10.1097/MD.0000000000022228

**Published:** 2020-09-18

**Authors:** Chunhoo Cheon, Seong-Gyu Ko

**Affiliations:** Department of Preventive Medicine, College of Korean Medicine, Kyung Hee University, Seoul, Republic of Korea.

**Keywords:** clinical trial, combination therapy, docetaxel, herbal medicine, phase 1 study, SH003, study protocol

## Abstract

**Introduction:**

Cancer is the second leading cause of death, and the burden of cancer continues to grow globally. Research on the efficacy of combined administration of herbal medicine and anticancer drugs is also increasing. SH003 is a new herbal medicine composed of *Astragalus membranaceus*, *Angelica gigas*, and *Trichosanthes kirilowii*. SH003 alone up to 4800 mg daily was found to be safe. Preclinical studies have shown SH003 to have a synergistic effect with coadministration of anticancer drugs. This study aimed to determine the maximum tolerated dose of SH003 combined with docetaxel in patients with lung or breast cancer.

**Methods:**

This is an open-label, dose-escalation study to evaluate the safety of SH003 combined with docetaxel. Patients with lung or breast cancer will be recruited. The participants will be divided into 3 groups based on SH003 daily dose (2400, 3600, and 4800 mg); the medication will be taken orally for 21 days. The traditional 3 + 3 design will be adopted for the dose escalation. Dose-limiting toxicities are defined as grade 3 or 4 adverse events according to the Common Terminology Criteria for Adverse Events. The highest dose at which no more than 1 of the 6 patients experience dose-limiting toxicity will be determined as the maximum tolerated dose of SH003 combined with docetaxel.

**Discussion:**

This study investigates the safety of SH003 when combined with docetaxel. The results of this study will provide a safe dose range for conducting therapeutic exploratory trials.

**Trial registrations:**

ClinicalTrials.gov NCT04360317.

## 1 Introduction

Cancer is the second leading cause of death, with an estimated 9.6 million deaths, or 1 in 6 deaths in 2018, and the global burden of cancer continues to grow.^[1]^ Cancer research is being actively conducted worldwide, and mutual cooperation between countries and investigators is becoming important.^[2]^ Several herbal medicines have received attention as novel anticancer agents, and studies on their mechanisms are also actively being conducted.^[3]^ Although not fully investigated, regulation of micro-ribonucleic acid and modulating tumor microenvironment were reported as the mechanisms of action of several herbal medicines.^[3–5]^ Several studies of combined administration with anticancer agents have been conducted, and results have been reported. Herbal medicine combined with chemotherapy was reported to be effective for treating fatigue in cancer patients.^[6]^ The survival rate of patients with gastric cancer using herbal medicines in combination with chemotherapy was higher.^[7]^ Herbal medicines combined with conventional treatment increased the number of natural killer cells in patients with cancer.^[8]^ Integrative herbal medicine reduced chemotherapy-induced peripheral neuropathy and hand-foot syndrome in patients with colorectal cancer.^[9]^

SH003 is a new herbal medicine composed of Huang-Qi (Astragalus membranaceus), Dang-Gui (Angelica gigas), and Gua-Lou-Gen (Trichosanthes kirilowii). According to the theory of Korean medicine, Huang-Qi can efficiently tonify qi, Dang-Gui can efficiently tonify blood, and Gua-Lou-Gen can efficiently disperse swelling and expel pus.^[10]^ SH003 has apoptotic and antiangiogenic efficacy, as proven through several in vitro and in vivo studies.^[11–16]^ A phase I study conducted to evaluate the maximum tolerated dose in humans, confirmed the daily safe dose to be up to 4800 mg.^[17,18]^ The various effects of SH003, including the anticancer effect of single administration, the synergistic effect in combination with chemotherapy, and the effect of alleviating the adverse effects of anticancer drugs, have been studied. Among them, studies on the synergistic effect of coadministration with doxorubicin and paclitaxel have been published.^[19,20]^ SH003 is also effective in coadministration with docetaxel, though the study has not been published yet.

Taxane-based anticancer drugs, such as paclitaxel, docetaxel, and abraxane, are antimicrotubule agents inhibiting the regeneration of the microtubule network, which is essential for cell function. However, only paclitaxel and docetaxel are used as adjuvants.^[21]^ Paclitaxel is used for Kaposi sarcoma, breast cancer, endometrial cancer, lung cancer, bladder cancer, and cervical carcinoma.^[22]^ Docetaxel is used for breast cancer, head and neck cancer, gastric cancer, ovarian cancer, and non-small cell lung cancer.^[23]^

Based on previous studies, we aimed to explore the efficacy and safety of the combination therapy of SH003 and docetaxel in patients with docetaxel indications, and we planned a phase I study prior to confirming the safe dose range of the combination therapy of SH003 and docetaxel.

## 2 Materials and methods

### 2.1 Study design

**Figure 1:**
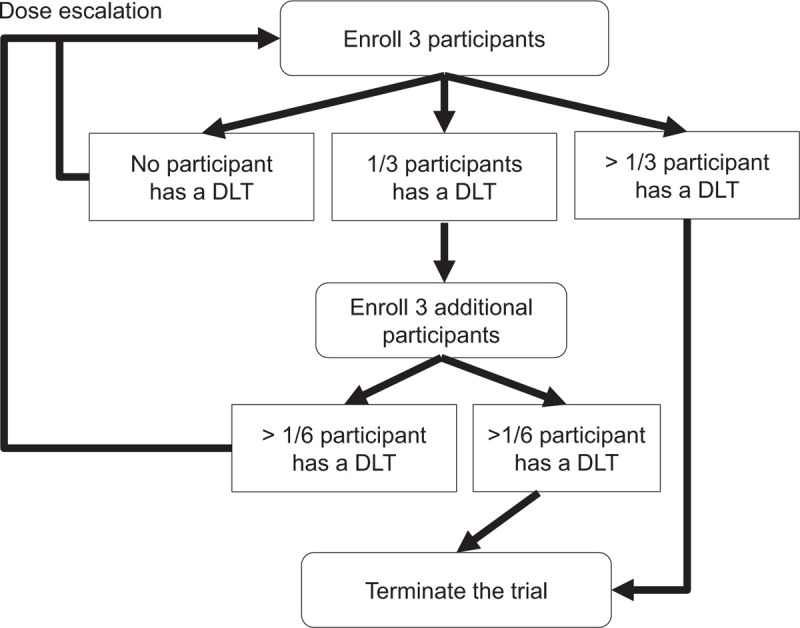
Study flow chart.

This study will be conducted at the Korea University Anam Hospital in Seoul and Ajou University Hospital in Suwon, Republic of Korea. Participants who meet the eligibility criteria will be enrolled. The enrolled participants will be assigned to 1 of the 3 groups receiving 2400, 3600, and 4800 mg daily doses of SH003. These doses represent the content of active ingredients found in a half of 1 tablet. Dose escalation is scheduled twice, following the modified Fibonacci sequence. The second dose will be 1.5 times the first dose and the third dose will be 1.33 times the second dose. Adverse events (AEs) of each participant will be assessed at each visit during the study period, for determination of increasing the dose and conducting the research. The schematic flow of this study is shown in Figure 1. Protocol amendments are not expected; however, if necessary, any changes in the protocol will be communicated to all investigators through a conference. This study was approved in May 2020 and is currently in progress.

### 2.2 Recruitment

Participants will be recruited primarily on the recommendation of a physician. Patients who visit the trial institution and meet the inclusion criteria will be recommended to participate in the present study by the physician. Detailed information such as the study period, purpose of the study, inclusion and exclusion criteria, and interventions will be communicated by the investigators.

### 2.3 Participants

#### 2.3.1 Inclusion criteria

1. Patients aged >19 years
2. Patients with histologically or cytologically confirmed lung or breast cancer for which standard curative measures do not exist or are no longer effective
3. Patients who have not received chemotherapy, radiotherapy, or surgery within the last 4 weeks and exhibit no residual toxicity associated with the previous treatment (Grade 1 or higher AEs according to Common Terminology Criteria for Adverse Events (CTCAE) ver 5.0 from the National Cancer Institute (NCI; Bethesda, MD))^[24]^
4. Eastern Cooperative Oncology Group Performance Status ≤ 2
5. Life expectancy estimated to be at least 12 weeks
6. Patients with the ability to swallow tablets
7. Patients with measurable lesions according to Response Evaluation Criteria in Solid Tumors version 1.1
8. Patients with proper organ function as follows
  i. Bone marrow function: hemoglobin ≥8 g/dL, absolute neutrophil count ≥1500/μL, and platelets ≥ 100,000/μL.
  ii. Liver function: total bilirubin, aspartate aminotransferase, or alanine aminotransferase ≤ 2.5 times the upper limit of normal (ULN) (if patients with liver metastasis, ≤5 times the ULN).
  iii. Renal function: Serum creatinine ≤1.5 times the ULN or creatinine clearance according to the Cockroft-Gault equation ≥ 60 mL/min
9. No possibility of pregnancy if the participant is female (over 60 years of age, without menstruation for more than 1 year, or underwent hysterectomy or bilateral oophorectomy). If there is a possibility of pregnancy, a pregnancy test should be conducted prior to participation in the study to determine pregnancy
10. Patients who agree to use effective means of contraception during the trial and up to 8 weeks after the final administration
11. Patients with the ability to understand the study and are willing to sign written informed consent document

#### 2.3.2 Exclusion criteria

1. Patients undergoing any systemic therapy or regional therapy including radiotherapy for treating cancer
2. Participants with known hypersensitivity to any study drug component, including *Astragalus membranaceus*, *Angelica gigas*, *Trichosanthes kirilowii, and polysorbate 80*
3. Patients with active infections requiring treatment (active hepatitis A, B, and C viruses, human immunodeficiency virus, tuberculosis)
4. History of human immunodeficiency virus infection
5. Patients with uncontrolled cardiovascular diseases (unstable angina, heart failure, myocardial infarction, uncontrolled hypertension: 140/90 mm Hg or higher)
6. Patients with active cytomegalovirus infection within the preceding 4 weeks
7. Patients with major surgery for cerebrovascular disease such as acute coronary syndrome, stroke, etc., within the past year
8. Pregnant or lactating females
9. Patients with metastatic encephalopathy with symptoms
10. Patients who have donated blood or participated in other clinical trials of medicine or medical devices within the past month
11. Patients who have undergone organ transplantation including allogeneic stem cell transplantation.
12. Patients with infectious diseases complications
13. Patients with suspected fever caused by infection
14. Patients with substance abuse or any neurological, medical, psychological, or sociological conditions potentially interfering with their compliance to the study protocol or interpretation of study results.
15. Patients judged inappropriate for the study by the investigator
16. Patients judged to have lost their ability to consent due to accompanying diseases such as dementia.

#### 2.3.3 Subject withdrawal criteria

1. Progression of disease despite administration of the drugs
2. Occurrence of uncontrolled AEs
3. Receiving other treatments for anticancer purposes
4. Participants’ withdrawal of consent
5. Occurrence of serious AEs related to the investigational drug
6. Significant protocol violations during the trial, including the detection of eligibility violations
7. Investigator's decision to terminate for participants’ health

If patients withdraw due to withdrawal of consent or the occurrence of a dose-limiting toxicity (DLT) not related to the investigational product, an additional number of participants will be recruited and replaced. Any loss caused by this clinical trial will be reimbursed by insurance.

### 2.4 Sample size

This study is a phase 1 study to evaluate the maximum tolerated dose (MTD) of SH003 combined with docetaxel for patients with solid cancers. This study was designed as a single group, dose-elevating trial evaluating safety to confirm the MTD by the combination of oral administration of SH003 to docetaxel patients. The traditional 3 + 3 design will be adopted for the dose escalation.^[25]^ First, 3 participants will be recruited and administered with the starting dose of 2400 mg/d for 21 days. If no DLT occurs, the dose will be increased to the second dose of 3600 mg/d. If DLT occurs, an additional 3 participants will be recruited and administered 2400 mg/d. If 2 or more of the 6 participants show DLT, the dose increase will be stopped, whereas if DLT occurs in less than 1 person, the dose will be increased to 3600 mg/d. Next, 3 participants will be recruited and administered with a dose of 3600 mg/d for 21 days. If no DLT occurs, the dose will be increased to 4800 mg/d. If DLT occurs, 3 participants will be additionally recruited and administered 3600 mg/d. Then, if 2 or more of the 6 participants show DLT, the dose increase will be stopped, whereas if DLT occurs in less than 1 person, the dose will be increased to 4800 mg/d. Finally, 3 participants will be recruited and administered with a dose of 4800 mg/d for 21 days. If no DLT occurs, the study is completed. If DLT occurs, an additional 3 participants will be recruited and administered 4800 mg/d and the study is therefore completed. Therefore, the sample size of the present study is a minimum of 3 participants and a maximum of 18. A total of 3 to 6 participants will be allocated to each dose of SH003.

### 2.5 Allocation

The enrolled participants will be assigned to the 2400 mg group, 3600 mg group, and 4800 mg group. After 3 participants in a group are recruited, recruitment will be stopped until an evaluation of the dose safety will be completed. Recruitment of additional participants or dose escalation will be determined by the safety evaluation result according to the occurrence of DLT.

### 2.6 Treatment protocol

The participants will receive SH003 combined with docetaxel for 21 days. They will orally take 2 to 4 tablets of SH003 with water thrice daily after meals for 21 days, depending on the group assigned. Docetaxel will be administered 75 mg/m^2^ intravenously for 1 hour on the first day of the study. In a 13-week repeated, oral toxicity study, no abnormal findings related to the investigational product were observed. No observed adverse effect level (NOAEL) of the investigational product is 2500 mg/kg among both sexes.^[11]^ When divided by human/animal factor, according to FDA guidelines, 2500 mg/kg NOAEL of a rat can be equated to 400 mg/kg in humans.^[26]^ When setting the safety factor at 10, the maximum recommended starting dose will be 40 mg/kg and it can be calculated as 2400 mg in a 60 kg human body. The participants will be required to return the remaining SH003 to calculate medication compliance. In principle, drugs other than the investigational products should not be administered for the purpose of anticancer treatment during the study period.

### 2.7 Interventions

One tablet of SH003 (total 800 mg) included 400 mg of solid extracts from *A Membranaceus*, *A Gigas*, and *T Kirilowii* (1:1:1) in a 30% ethanol extract. Hanpoong Pharm and Foods Co Ltd, the pharmaceutical company (Jeonju, Republic of Korea.) produced SH003 according to Korea good manufacturing practice standards.

### 2.8 Primary outcome measurement

The primary outcome measurement of this study will be DLT, defined as grade 3 or 4 according to the NCI CTCAE v5.0. CTCAE is a list of AE terminology for AE reporting, and includes a grading scale for each AE term. Trained investigators will measure the AEs at every visit. The details of the DLTs noted in this study are as follows. Hematologic toxicity: grade 4 neutropenia lasting more than 7 days, febrile neutropenia over grade 3, neutropenic infection of grade 3 or higher, thrombocytopenia of grade 3 or higher with bleeding, or grade 4 without bleeding. Nonhematologic toxicity: grade 3 or higher nausea, vomiting, and diarrhea despite treatment at the highest dose, grade 3 or higher liver toxicity or liver function abnormality, if the drug-related toxicity in the first cycle of administration continues and the next administration is postponed for more than 14 days.

### 2.9 Secondary outcome measurement

Secondary outcome measurements include AEs, irrespective of grade, during the trial, as measured by the NCI CTCAE v5.0. The study schedule is detailed in Table 1.

**Table 1:**
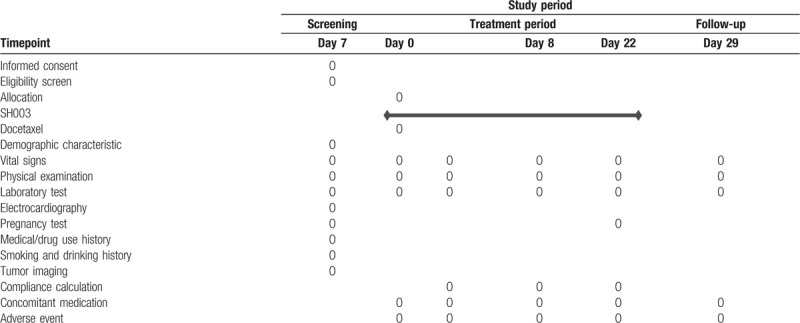
Study schedule of SH003 + Docetaxel Phase 1 study.

### 2.10 Outcomes analysis

#### 2.10.1 Determination of the maximum tolerated dose

For each dose group, the number of participants with AEs corresponding to grade 3 or higher based on CTCAE v. 5.0 will be evaluated. If there are 1 or fewer participants who have an adverse event corresponding to grade 3 or higher, the dose will be considered a safe dose. The highest dose found safe in the present study will be determined as the MTD of SH003 when combined with docetaxel.

The results of this study will be presented as a descriptive analysis. The continuous variables will be presented as the median and range, and the categorical variables will be presented as absolute and relative frequencies. Each participant's occurrence of DLT will be evaluated immediately and the result will determine whether or not the next dose will proceed.

### 2.11 Data and safety monitoring

For the quality of this study, the Contract Research Organization will conduct monitoring. Institutions participating in this trial will be monitored by standard operating procedures during the study period. Double data entry will be performed, and range check will be performed on the dataset, to improve the quality of data. If suspected and unexpected serious adverse reactions occur, it will be reported to the institutional review board (IRB) and the Ministry of Food and Drug Safety (MFDS) in the Republic of Korea. Participants withdrawn due to AEs will be followed up till they recover from the AEs.

### 2.12 Ethics and dissemination

This study has been approved by the IRBs of the Korea University Anam Hospital (2020AN0020), and Ajou University Hospital (AJIRB-MED-T12-19-479). The current protocol version is 1.3. Investigators will obtain written informed consent from each participant prior to the initiation of the clinical trial. This study will be conducted in compliance with the Helsinki Declaration and according to Good Clinical Practice as described by the MFDS. Participants will be identified with the study identification number assigned at the beginning of the study to protect confidentiality. All clinical trial documents will be stored in locked cabinets or password-protected computer files. Only authorized researchers can access the data. The results of the trial will be communicated via publication in a Scientific Journal or Conference.

## 3 Discussion

When cancer patients are treated with chemotherapy, they often do not undergo planned chemotherapy due to toxicity. Docetaxel has also reported various adverse reactions such as neutropenia, leukopenia, thrombocytopenia, anemia, infection, fluid retention, diarrhea, vomiting, and asthenia.^[27]^ If these toxicities can be alleviated using herbal medicine, the scheduled anticancer therapy can be completed and the patient's quality of life and health can be greatly improved. In China, many studies have been conducted to classify the pattern differentiation of various cancers according to their symptoms, and a guideline has been developed to describe herbal medicine for each pattern differentiation.^[28,29]^ In many countries, herbal medicine is used as an adjuvant treatment for anticancer therapy.^[30]^ In the United States, research has been conducted to develop herbal medicine reducing the adverse reactions to anticancer drugs and improve the anticancer efficacy.^[31,32]^ In Korea, studies were conducted to evaluate the efficacy of herbal medicines on fatigue, radiation dermatitis, sleep disturbance, anorexia, and cough in patients with cancer.^[33–38]^ These studies used licensed herbal medicines for patients with cancer. This study is a clinical trial of a new herbal medicine for the treatment of cancer patients, which has been rarely performed in Korea.

In the present study, we will evaluate the safe dose range for SH003 combined with docetaxel. The dose of SH003 up to 4800 mg/d is considered safe when administered alone, but the starting dose of the present study is 2400 mg as it is the first clinical trial of combination therapy with anticancer agents. SH003 has a possibly synergistic effect on anticancer efficacy in combination with anticancer agents and reduces their adverse reactions.

The limitation of the present study is that although various anti-cancer agents are used, only docetaxel was selected as a combination drug. This was determined by considering the results of preclinical studies supporting the clinical trial and the feasibility of the clinical trial. In the future, clinical studies for the combined administration of various anticancer agents are being planned.

Although there are a few limitations, this study has the following features. This study is the first combination clinical trial conducted with the SH003. In Korea, most herbal medicine studies in patients with cancer have used licensed herbal medicines, but this study is about a new herbal medicine. Therefore, this study can be considered a guide for new herbal medicine studies considering concomitant administration with anticancer agents.

## Author contributions

**Conceptualization:** Seong-Gyu Ko.

**Funding acquisition:** Seong-Gyu Ko.

**Methodology:** Chunhoo Cheon.

**Supervision:** Seong-Gyu Ko.

**Writing – original draft:** Chunhoo Cheon.

**Writing – review & editing:** Chunhoo Cheon, Seong-Gyu Ko.

## Abbreviations

Abbreviations: AEs = adverse events, CTCAE = Common Terminology Criteria for Adverse Events, DLT = dose-limiting toxicity, IRB = Institutional Review Board, MFDS = Ministry of Food and Drug Safety, MTD = maximum tolerated dose, NCI = National Cancer Institute, NOAEL = no observed adverse effect level, ULN = upper limit of normal.
